# LDH/MXene Synergistic Carrier Separation Effects to Improve the Photoelectric Catalytic Activities of Bi_2_WO_6_ Nanosheet Arrays

**DOI:** 10.3390/nano14050477

**Published:** 2024-03-06

**Authors:** Yuting Wang, Runhua Li, Jiaying Zhang, Liming Liu, Weiwei Huang, Yajun Wang

**Affiliations:** State Key Laboratory of Heavy Oil Processing, China University of Petroleum, Beijing 102249, China; wangyt@cup.edu.cn (Y.W.); 2021215954@student.cup.edu.cn (R.L.); 2020211160@student.cup.edu.cn (J.Z.); 2021211158@student.cup.edu.cn (L.L.); 2022211152@student.cup.edu.cn (W.H.)

**Keywords:** Bi_2_WO_6_, MXene, NiFe-LDH, photoelectrocatalytic, bisphenol A

## Abstract

Photoelectric catalysis is a green and efficient way to degrade pollutants, which has been paid more and more attention by researchers. Among them, Bi_2_WO_3_ has been proved to have excellent photocatalytic oxidation activity on its {001} facets. In this study, {001}-oriented facets with high exposure were successfully integrated into Bi_2_WO_6_ nanoplate arrays (Bi_2_WO_6_ NAs) to create a photoelectrode. This structure was grown in situ on an indium tin oxide (ITO) substrate. To promote photogenerated carrier separation efficiency and reduce agglomeration of Bi_2_WO_6_ photocatalysts, the electrochemical deposition of NiFe–layered double hydroxide (NiFe-LDH) and Ti_3_C_2_ (MXene) were introduced in this research to synergistically catalyze pollutant degradation. Morphology, spectral characterization, and electrochemical analysis jointly confirmed that the outstanding performance of hole capture behavior with LDH and electron conduction properties with MXene were the main reasons for the improvement in catalytic activity of the photoelectrode. Taking bisphenol A (BPA) as the model pollutant, the rate constant *k* of the NiFe-LDH/Ti_3_C_2_/Bi_2_WO_6_ NAs photoelectrode reaches 0.00196 min^−1^ under photoelectrocatalytic (PEC) conditions, which is 4.5 times that of the pure Bi_2_WO_6_ NAs photoelectrode. This work provides a new way to improve the reaction kinetics of the PEC degradation of pollutants.

## 1. Introduction

Photoelectrocatalysis, as a green way to solve water pollution, has the advantages of electrocatalytic (EC) and photocatalytic (PC) oxidation [1,2]. During the photoelectrocatalytic (PEC) process, photogenerated electrons are driven towards the counter electrode by an external potential. This mechanism facilitates the efficient separation of photogenerated electrons and holes, thereby significantly enhancing photocatalytic activity [3,4,5,6].

As a significant semiconductor photocatalytic material, Bi_2_WO_6_ is the simplest compound in the Aurivillius family, which is alternately composed of [Bi_2_O_2_]^2+^ layers and perovskite-like (WO_4_)^2−^ octahedral layers, and is widely used in PEC oxidation [3,7]. As a narrow band gap semiconductor (2.75 eV), Bi_2_WO_6_ has the advantages of a non-toxic, high-stability, and visible-light response [8,9,10]. Nevertheless, this material exhibits certain drawbacks, such as a high rate of electron and hole recombination, a low specific surface area, and a tendency towards easy agglomeration [4,11,12]. In order to address these issues, a variety of strategies have been employed to improve the photocatalytic performance of Bi_2_WO_6_. These methods include doping, the construction of heterojunctions, and surface hybridization [8,13,14,15]. In 2005, Zhu and co-workers synthesized Bi_2_WO_6_ nanostructures for the first time through simple hydrothermal treatment, which set a precedent for the preparation of Bi_2_WO_6_ nanostructures [16]. Cao and co-workers successfully prepared the 2D/2D heterojunction of ultra-thin Ti_3_C_2_/Bi_2_WO_6_ nanosheets, which have a short charge transfer distance and large interface contact area. Under simulated solar light irradiation, the combined yield of CH_4_ and CH_3_OH achieved by the Ti_3_C_2_/Bi_2_WO_6_ composite is 4.6 times higher than that of pure Bi_2_WO_6_ in the CO_2_ reduction reaction [17].

Layered double hydroxide (LDH) is a type of layered inorganic functional material, characterized by a positively charged layer and an anion that is exchangeable in the interlayer space [18,19,20]. LDH has attracted much attention due to its unique layered structure and exchangeable anion between layers [21,22]. In recent years, Zhu et al. reported three-dimensional BiVO_4_/NiFe-LDH heterostructures. The combination of BiVO_4_ and NiFe-LDH can accelerate photogenerated charge separation and photoelectrocatalytic activity [23]. Zhang and colleagues synthesized a ZnCr–CO_3_LDH/ruptured tubular g-C_3_N_4_ photocatalyst and observed that its capability to separate electron–hole pairs was enhanced by approximately fivefold and twofold, respectively, compared to bulk g-C3N4 and ruptured tubular g-C_3_N_4_ [24]. MXenes, a novel class of two-dimensional (2D) layered materials, are composed of transition metal carbides or carbonitrides. The general formula for MXenes is M_n+1_X_n_ (where n = 1, 2, or 3), with ‘M’ representing a transition metal (such as Sc, Ti, Zr, Hf, V, Nb, Ta, or Mo), and ‘X’ denoting carbon (C) and/or nitrogen (N). Notably, MXenes (Ti_3_C_2_), synthesized by selectively etching out the ‘A’ layers from Ti_3_AC_2_ (where ‘A’ can be Al, Zn, Si, or Ga), have become a focal point in research on the synthesis of MXene-based photocatalysts [25,26]. It is mainly attributed to the following excellent properties of Ti_3_C_2_: (i) The Ti_3_C_2_ surface of MXenes, adorned with various terminal functional groups such as -OH, -O, and -F, can establish a strong chemical interface with semiconductors [27]. This interaction significantly contributes to inhibiting the recombination of electrons and holes [17]. (ii) The notable metallic conductivity of Ti_3_C_2_ ensures rapid carrier migration, thereby facilitating efficient separation of electrons and holes [28,29]. (iii) The exposed terminal metal sites on Ti_3_C_2_ are likely to result in higher reactivity compared to that of carbon-based materials [26,30]. Given the aforementioned characteristics, Ti_3_C_2_ has emerged as a favored co-catalyst in the synthesis of photocatalysts [31]. Li et al. reported that mesoporous TiO_2_ nanoparticles were successfully anchored onto a highly conductive Ti_3_C_2_ MXene co-catalyst using an electrostatic self-assembly strategy. The composite material, TiO_2_/Ti_3_C_2_, has demonstrated superior photocatalytic performance. This is particularly evident in its ability to degrade methyl orange, where it achieved an exceptionally high efficiency of 99.6%. Additionally, the material exhibits a significant hydrogen production rate, quantified at 218.85 μmol·g^−1^·h^−1^. These findings underscore the composite’s potential efficacy in photocatalytic applications [32]. Ran et al. synthesized Ti_3_C_2_ nanoparticles with CdS to induce a photocatalytic hydrogen production activity of 14,342 μmol·g^−1^·h^−1^. The enhanced performance observed can be attributed to the advantageous characteristics of the Ti_3_C_2_ nanoparticles. Specifically, favorable positioning of the Fermi level, coupled with the nanoparticles’ excellent electrical conductivity, play critical roles in facilitating the high efficiency of the composite. These properties significantly contribute to the overall superior photocatalytic performance of the material [28].

In this work, we synthesized a Bi_2_WO_6_ photoelectrode with a large proportional exposure of {001} facets on an ITO substrate in situ (Bi_2_WO_6_ NAs). Subsequently, the NiFe-LDH/Ti_3_C_2_/Bi_2_WO_6_ NAs photoelectrode was synthesized via electrochemical deposition, layering NiFe-LDH and delaminating Ti_3_C_2_ onto Bi_2_WO_6_ NAs. Delaminated MXenes were utilized due to their superior electrical conductivity and robust mechanical properties, which are essential for the photocatalytic applications investigated. The layered structure facilitates efficient electron transport and provides numerous active sites for photocatalytic reactions, thereby enhancing the overall performance of the photocatalysts. This approach ensured precise material integration for the composite electrode. Taking the degradation of bisphenol A (BPA) as an example, the effect of the combination of Ti_3_C_2_ and NiFe-LDH on the catalytic performance of the Bi_2_WO_6_ NAs photoelectrode was investigated. Under PC, EC, and PEC conditions, the degradation activity of the NiFe-LDH/Ti_3_C_2_/Bi_2_WO_6_ NAs photoelectrode is better than that of the Bi_2_WO_6_ NAs photoelectrode, and the order of activity from high to low is NiFe-LDH/Ti_3_C_2_/Bi_2_WO_6_ NAs > NiFe-LDH/Bi_2_WO_6_ NAs > Ti_3_C_2_/Bi_2_WO_6_ NAs > Bi_2_WO_6_ NAs. The extensive exposure of {001} facets in the Bi_2_WO_6_ nanoplate arrays (NAs) photoelectrode is advantageous for photocatalytic oxidation reactions. Moreover, the synergistic interaction between NiFe–layered double hydroxide (LDH) and Ti_3_C_2_ significantly enhances the separation efficiency of photogenerated carriers in Bi_2_WO_6_. Performance of the NiFe-LDH/Bi_2_WO_6_ photoelectrode and the mechanism of enhancing its PEC activity have been systematically studied.

## 2. Results and Discussion

### 2.1. Catalyst Characterization

**Figure 1:**
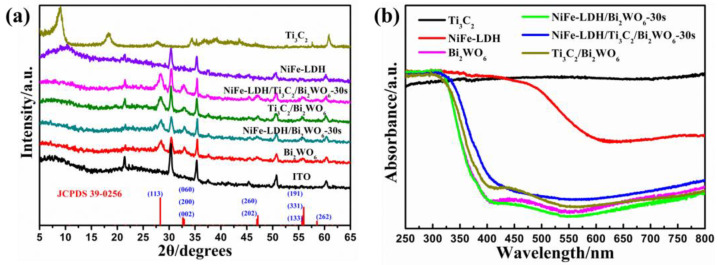
(**a**) XRD patterns of ITO, NiFe-LDH, Ti_3_C_2_, Bi_2_WO_6_ NAs, NiFe-LDH/Bi_2_WO_6_ NAs, Ti_3_C_2_/Bi_2_WO_6_ NAs, and NiFe-LDH/Ti_3_C_2_/Bi_2_WO_6_ NAs; (**b**) UV-Vis DRS spectra of NiFe-LDH, Ti_3_C_2_, Bi_2_WO_6_ NAs, NiFe-LDH/Bi_2_WO_6_ NAs, Ti_3_C_2_/Bi_2_WO_6_ NAs, and NiFe-LDH/Ti_3_C_2_/Bi_2_WO_6_ NAs.

The crystallographic structure and compositional analysis of the synthesized samples were examined utilizing X-ray diffraction (XRD) techniques. (Figure 1a). The XRD pattern shows three diffraction peaks at 8.9°, 18.3°, and 60.8°, corresponding to the (002), (004), and (110) planes of Ti_3_C_2_, respectively [29]. In addition, all of the diffraction peaks match well with the Bi_2_WO_6_ NAs according to JCPDS 39-0256 [33]. The observed intensity ratio of the {002} and {131} peaks in Bi_2_WO_6_ nanoplate arrays (NAs) is approximately 0.5, which is notably higher than the standard value of 0.19. This indicates that the as-prepared Bi_2_WO_6_ NAs exhibit anisotropic growth along the {001} planes. The extensive exposure of {001}-oriented Bi_2_WO_6_ NAs is advantageous, as it facilitates photocatalytic oxidation reactions [34]. After adding Ti_3_C_2_ (MXene) in the formation of Bi_2_WO_6_ NAs, the area integral ratio of diffraction peak between {002} and {131} crystal planes increases to 0.54, indicating that the existence of MXene expanded the {001}-orientated crystal faces exposure of Bi_2_WO_6_ NAs. The observed negativity of the Ti_3_C_2_ surface can be attributed to the adsorption of a substantial quantity of -F and -OH groups. This characteristic is particularly relevant during the preparation of MXene, where Bi^3+^ ions are adsorbed onto the Ti_3_C_2_ surface, further influencing its chemical behavior and interactions. Therefore, Bi_2_WO_6_ NAs expose more [Bi_2_O_2_]^2+^ layers parallel to the {001} crystal plane. In addition, the diffraction peak of Bi_2_WO_6_ NAs becomes wider, which is due to the growth-limiting effect of MXene on Bi_2_WO_6_ NAs [17]. UV-Vis Diffuse Reflectance Spectroscopy (DRS) spectra provide insights into the optical properties of the as-prepared samples. As shown in Figure 1b, Bi_2_WO_6_ NAs, NiFe-LDH/Bi_2_WO_6_ NAs, Ti_3_C_2_/Bi_2_WO_6_ NAs, and NiFe-LDH/Ti_3_C_2_/Bi_2_WO_6_ NAs photoelectrodes have obvious absorption responses in the visible light region. Moreover, the absorption edge of Ti_3_C_2_/Bi_2_WO_6_ NAs and NiFe-LDH/Ti_3_C_2_/Bi_2_WO_6_ NAs photoelectrodes is near 450 nm, indicating that the combination of Ti_3_C_2_ broadens the response of Bi_2_WO_6_ NAs to visible light.

**Figure 2:**
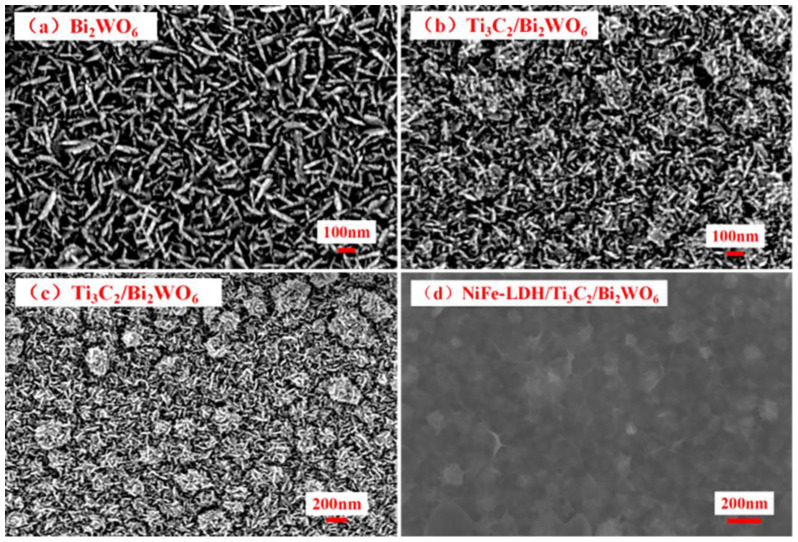
SEM images of (**a**) Bi_2_WO_6_ NAs; (**b**,**c**) Ti_3_C_2_/Bi_2_WO_6_ NAs; (**d**) NiFe-LDH/Ti_3_C_2_/Bi_2_WO_6_ NAs.

Scanning electron microscopy (SEM) images (Figure 2a and Figure S1) exhibit that Bi_2_WO_6_ nanosheets are vertically grown on the ITO substrate with a size of about 200 nm. The nanoplates are interconnected, forming a framework array structure. Following compounding with Ti_3_C_2_, the size of Bi_2_WO_6_ nanosheets is observed to be approximately 100 nm. This dimension is attributed to the growth restriction imposed by Ti_3_C_2_ on Bi_2_WO_6_. (Figure 2b,c) [17]. Figure 2d displays the structural morphology of the NiFe–layered double hydroxide (LDH)/Ti_3_C_2_/Bi_2_WO_6_ nanoplate arrays (NAs) photoelectrode. NiFe-LDH is observed to be uniformly coated on the Ti_3_C_2_/Bi_2_WO_6_ surface, presenting a flocculent morphology that is evenly dispersed. As shown in Supplementary Figure S2, the uniform detection of elements such as tungsten (W), bismuth (Bi), oxygen (O), nickel (Ni), iron (Fe), titanium (Ti), and carbon (C) confirms the even distribution of NiFe-LDH and Ti_3_C_2_ across the Bi_2_WO_6_ NAs.

### 2.2. Enhanced PEC Degradation Activity

**Figure 3:**
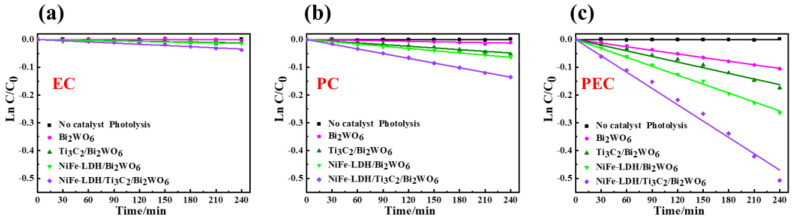
Comparison of (**a**) EC, (**b**) PC, and (**c**) PEC degradation over time, and its first-order reaction fitting curves over Bi_2_WO_6_ NAs, NiFe-LDH/Bi_2_WO_6_ NAs, Ti_3_C_2_/Bi_2_WO_6_ NAs, and NiFe-LDH/Ti_3_C_2_/Bi_2_WO_6_ NAs photoelectrodes (λ ≥ 420 nm, external potential = 1.0 V). The associated rate constant, denoted as *k*.

**Figure 4:**
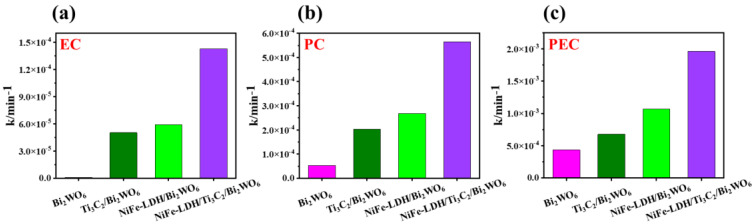
Comparison of (**a**) EC, (**b**) PC, and (**c**) PEC degradation rates over Bi_2_WO_6_ NAs, NiFe-LDH/Bi_2_WO_6_ NAs, Ti_3_C_2_/Bi_2_WO_6_ NAs, and NiFe-LDH/Ti_3_C_2_/Bi_2_WO_6_ NAs photoelectrodes.

Catalytic performance of as-prepared catalysts is estimated by the degradation of BPA in aqueous solution under PC, EC, and PEC systems. As can be seen from Figure S3, BPA does not undergo self-degradation under visible light irradiation. The EC activity of the Bi_2_WO_6_ NAs photoelectrode also shows a low degradation rate of BPA (Figure 3a and Figure 4a). Generally speaking, Ti_3_C_2_ and NiFe-LDH are excellent electrocatalytic materials with high conductivity, so the EC activity is significantly improved after compounding with Ti_3_C_2_ and NiFe-LDH. Upon exposure exclusively to visible light, the degradation rate constant, denoted as *k*, of the NiFe-LDH/Ti_3_C_2_/Bi_2_WO_6_ nanocomposite photoelectrode exhibits an enhancement by a factor of approximately 10 compared to the degradation rate constant of the pristine Bi_2_WO_6_ nanocomposite photoelectrode (Figure 3b and Figure 4b) due to the excellent electron transport properties of Ti_3_C_2_ and the fast trapping of photogenerated holes by NiFe-LDH. In the PEC system, the catalytic performance of Bi_2_WO_6_ NAs, Ti_3_C_2_/Bi_2_WO_6_ NAs, NiFe-LDH/Bi_2_WO_6_ NAs, and NiFe-LDH/Ti_3_C_2_/Bi_2_WO_6_ NAs photoelectrodes is further improved, all of which are higher than the PC or EC system alone (Figure 3c and Figure 4c). The external potential can quickly transfer the photogenerated electrons generated by Bi_2_WO_6_ NAs to the counter electrode through the external circuit. Consequently, PEC activity escalates with the augmentation of external potential. At external potentials below the water decomposition threshold, such potential facilitates the migration of electrons towards the counter electrode, thereby enhancing the segregation of photogenerated electrons and holes. Conversely, when the external potential surpasses the threshold for water decomposition, it instigates complex oxidation processes. Figure S4 shows the PEC performance of the NiFe-LDH/Ti_3_C_2_/Bi_2_WO_6_ NAs photoelectrode under different external potentials. Under the irradiation of visible light (λ ≥ 420 nm), the PEC degradation rate of BPA gradually increases with increasing external potential. However, when the external potential reaches 3.0 V, the surface of the Bi_2_WO_6_ NAs photoelectrode blackens rapidly, leading to deactivation of the catalyst and a sharp decrease in degradation activity.

### 2.3. Enhancement Mechanism of Photoelectrochemical (PEC) Activity

**Figure 5:**
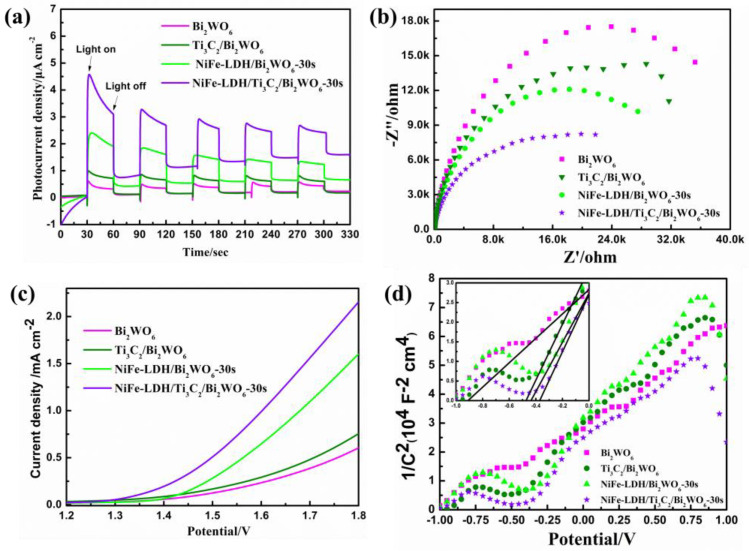
(**a**) Transient photocurrent density; (**b**) EIS Nyquist plots; (**c**) linear sweep voltammetry plots; (**d**) Mott–Schottky plots of Bi_2_WO_6_ NAs, NiFe-LDH/Bi_2_WO_6_ NAs, Ti_3_C_2_/Bi_2_WO_6_ NAs, and NiFe-LDH/Ti_3_C_2_/Bi_2_WO_6_ NAs (λ ≥ 420 nm).

To elucidate the underlying mechanism behind the augmented PEC activity of NiFe-LDH/Ti_3_C_2_/Bi_2_WO_6_ nanocomposites, an array of photoelectrical characterizations has been conducted. As can be seen from Figure 5a, the photocurrent density of the NiFe-LDH/Ti_3_C_2_/Bi_2_WO_6_ NAs photoelectrode reaches about 3.0 μA/cm^2^, which represents an enhancement of approximately 3.8-fold compared to the Ti_3_C_2_/Bi_2_WO_6_ nanocomposite photoelectrode and a 6.0-fold increase over the pristine Bi_2_WO_6_ nanocomposite photoelectrode. The increase of photocurrent density of the NiFe-LDH/Ti_3_C_2_/Bi_2_WO_6_ NAs photoelectrode is attributed to the synergistic effect of NiFe-LDH and Ti_3_C_2_, which greatly improves the separation efficiency of Bi_2_WO_6_ NAs photogenerated charge. Electrochemical impedance spectroscopy is utilized to investigate the impedance characteristics of the synthesized samples, as depicted in Figure 5b. The curve radius of the pure Bi_2_WO_6_ NAs photoelectrode is the largest, and the impedance value is also the largest. After compounding with Ti_3_C_2_ and NiFe-LDH, the curve radius of the NiFe-LDH/Ti_3_C_2_/Bi_2_WO_6_ NAs photoelectrode decreases and the impedance value decreases accordingly, which is consistent with the above transient photocurrent response results. The catalytic performance of the as-prepared sample is investigated by linear sweep voltammetry (LSV) (Figure 5c). The order of current density under different external potentials is NiFe-LDH/Ti_3_C_2_/Bi_2_WO_6_ NAs > NiFe-LDH/Bi_2_WO_6_ NAs > Ti_3_C_2_/Bi_2_WO_6_ NAs > Bi_2_WO_6_ NAs. Furthermore, the starting potential of the NiFe-LDH/Ti_3_C_2_/Bi_2_WO_6_ NAs photoelectrode has an obvious negative shift, indicating that the NiFe-LDH/Ti_3_C_2_/Bi_2_WO_6_ NAs photoelectrode has a stronger redox ability. From Figure 5d, the curve slopes of the Bi_2_WO_6_ NAs, NiFe-LDH/Bi_2_WO_6_ NAs, Ti_3_C_2_/Bi_2_WO_6_ NAs, and NiFe-LDH/Ti_3_C_2_/Bi_2_WO_6_ NAs photoelectrodes are all positive, which demonstrates that Bi_2_WO_6_ is an N-type semiconductor. At the same time, after compounding with Ti_3_C_2_ and NiFe-LDH, the flat band potential shifts significantly, illustrating that the band edge bending of the NiFe-LDH/Ti_3_C_2_/Bi_2_WO_6_ NAs photoelectrode decreases. It is beneficial to promote charge transfer at the electrode/electrolyte interface, accelerate the surface reaction kinetics process, and then enhance catalytic performance. To investigate the correlation between the photoelectric properties and light absorption capabilities of the synthesized samples, the photoelectric conversion efficiency of Bi_2_WO_6_ NAs, NiFe-LDH/Bi_2_WO_6_ NAs, Ti_3_C_2_/Bi_2_WO_6_ NAs, and NiFe-LDH/Ti_3_C_2_/Bi_2_WO_6_ NAs photoelectrodes are calculated (Figure S5). All samples have a photoelectric conversion ability in the ultraviolet region, and the NiFe-LDH/Ti_3_C_2_/Bi_2_WO_6_ NAs photoelectrode has the strongest photoelectric conversion ability.

**Figure 6:**
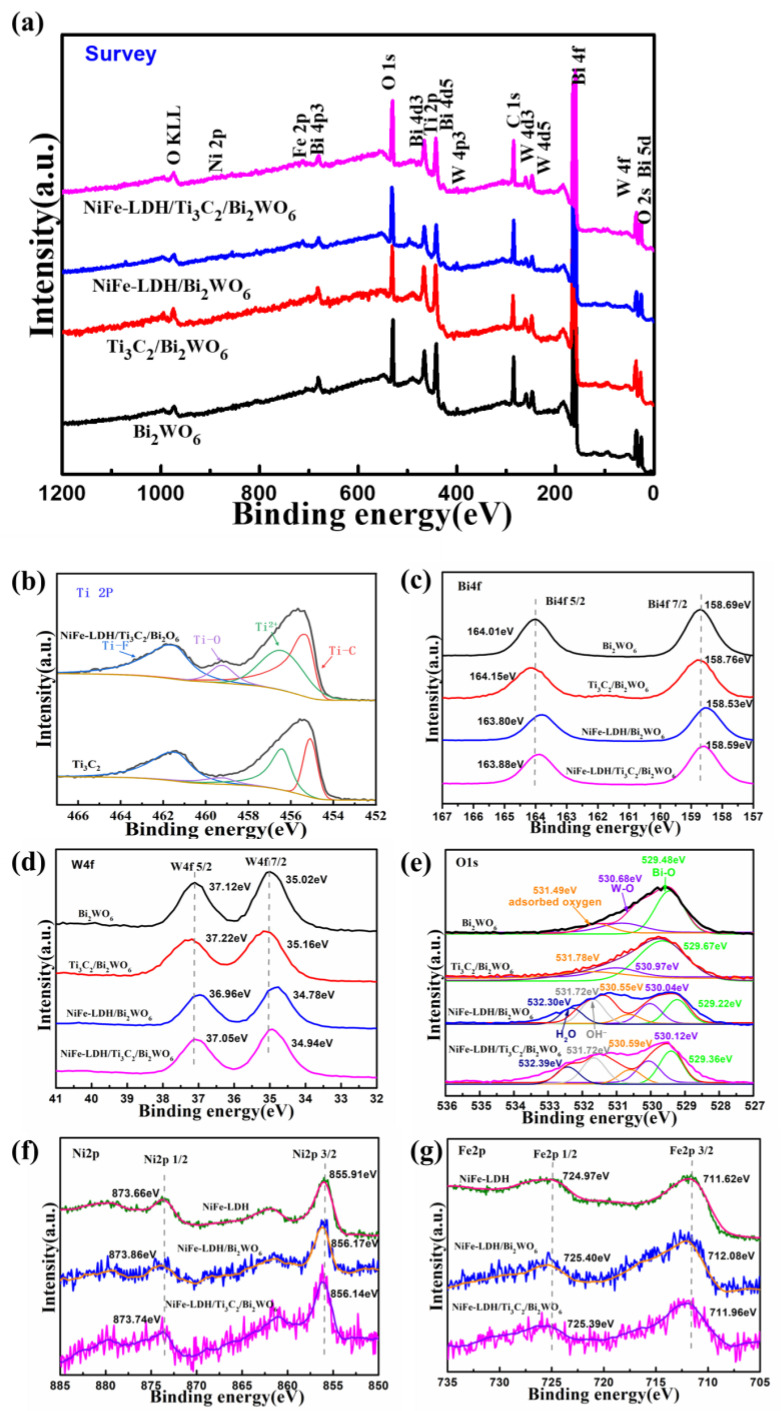
XPS of (**a**) survey spectra, (**b**) Ti 2p, (**c**) Bi 4f, (**d**) W 4f, (**e**) O 1s, (**f**) Ni 2p, and (**g**) Fe 2p of NiFe-LDH, Bi_2_WO_6_ NAs, NiFe-LDH/Bi_2_WO_6_ NAs, Ti_3_C_2_/Bi_2_WO_6_ NAs, and NiFe-LDH/Ti_3_C_2_/Bi_2_WO_6_ NAs.

Figure 6 presents the outcomes of X-ray photoelectron spectroscopy (XPS) analyses performed on the catalysts. It was found that Bi, W, O, Ni, Fe, Ti, and C elements are present in the NiFe-LDH/Ti_3_C_2_/Bi_2_WO_6_ NAs photoelectrode (Figure 6a). Figure 6b illustrates the predominance of Ti-C bonds within the Ti2p peaks, alongside a minor presence of Ti-O structures. This suggests that while there may be slight oxidation of the Ti_3_C_2_ material, it does not detract from the dominant influence of the Ti_3_C_2_ structure in the ternary catalytic system. Figure 6c,d show the chemical states of Bi 4f and W 4f of as-prepared samples. Compared with that of Bi_2_WO_6_ NAs, the binding energy of Ti_3_C_2_/Bi_2_WO_6_ NAs in Bi 4f and W 4f spectra show positive shifts. However, the peak shifts of Bi 4f and Bi 4f_5/2_ in the NiFe-LDH/Bi_2_WO_6_ NAs photoelectrode are opposite [13,35]. Moreover, the O 1s spectra of the as-prepared sample in Figure 6e consists of Bi-O, W-O, and surface adsorbed oxygen [36]. The peak positions of Bi-O and W-O in the Ti_3_C_2_/Bi_2_WO_6_ NAs photoelectrode show positive shifts, and the position of adsorbed oxygen shifts to 531.78 eV. On the contrary, the peak positions of Bi-O and W-O in the NiFe-LDH/Bi_2_WO_6_ NAs photoelectrode shift negatively to 529.22 eV and 530.04 eV, and the peaks at 530.55 eV, 531.72 eV, and 532.30 eV belong to surface adsorbed oxygen, interlayer anion (OH^−^), and H_2_O of NiFe-LDH, respectively. The above proves that Bi_2_WO_6_ NAs interact with Ti_3_C_2_ and NiFe-LDH, respectively, inducing the migration of photogenerated electrons from Bi_2_WO_6_ NAs to Ti_3_C_2_ and photogenerated holes from Bi_2_WO_6_ NAs to NiFe-LDH. Furthermore, an upward shift in the peak positions of Ni 2p and Fe 2p observed in NiFe-LDH/Bi_2_WO_6_ nanocomposite photoelectrodes and NiFe-LDH/Ti_3_C_2_/Bi_2_WO_6_ nanocomposite photoelectrodes (as shown in Figure 6f,g) corroborates the interaction between the surface energy of Bi_2_WO_6_ nanocomposites and NiFe-LDH. This charge transfer mechanism aids in the efficient separation of electrons and holes, thereby improving photoelectrochemical performance.

**Figure 7:**
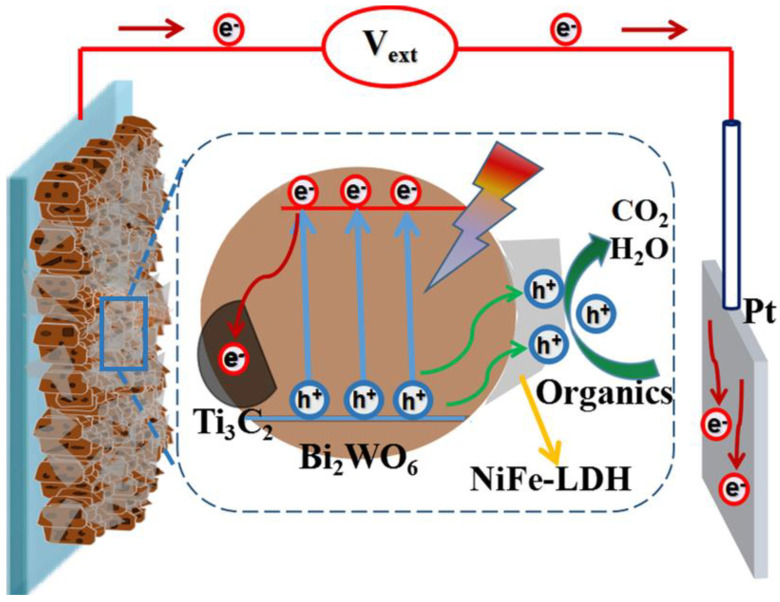
Schematic representation illustrating the photoelectrocatalytic degradation mechanism of organic compounds via the NiFe-LDH/Ti_3_C_2_/Bi_2_WO_6_ nanocomposite photoelectrode. This diagram delineates the process of photogenerated electron–hole pair separation and subsequent transfer, highlighting the synergistic effects of the composite materials in enhancing the degradation efficiency of organic pollutants (The red and green arrows indicate the direction of movement of electrons and holes, respectively).

Building on the preceding discussion, a more detailed mechanism is proposed for the photoelectrochemical (PEC) degradation of organic pollutants using the NiFe-LDH/Ti_3_C_2_/Bi_2_WO_6_ nanocomposite photoelectrode, as illustrated in Figure 7. The ternary composite system comprising Ti_3_C_2_, Bi_2_WO_4_, and layered double hydroxide (LDH) forms a unique and sophisticated heterojunction that exhibits characteristics of both Type-II and Z-scheme heterojunctions. This innovative configuration leverages the distinct electronic properties of each component to facilitate efficient charge separation and transfer, thereby enhancing photocatalytic performance under light irradiation. The Ti_3_C_2_/Bi_2_WO_4_ interface forms a Type-II heterojunction, where alignment of their conduction and valence bands allows for the spatial separation of photogenerated electrons and holes. Electrons tend to migrate towards Ti_3_C_2_, while holes accumulate in Bi_2_WO_4_, thus reducing the recombination rate and enhancing photocatalytic efficiency. The addition of LDH into the Ti_3_C_2_/Bi_2_WO_4_ system introduces a Z-scheme mechanism, particularly when LDH acts as a bridge for electron transfer between Ti_3_C_2_ and Bi_2_WO_4_. This configuration preserves the high reduction potential of Ti_3_C_2_’s electrons and the high oxidation potential of Bi_2_WO_4_’s holes, making the composite highly effective for redox reactions.

The LDH/Ti_3_C_2_/Bi_2_WO_6_ ternary composite system leverages a unique combination of Type-II and Z-scheme heterojunctions, offering significant advantages for photocatalytic applications. This sophisticated heterostructure ensures enhanced charge separation and transfer, effectively minimizing recombination and maximizing the availability of reactive charge carriers for photocatalysis. Additionally, the incorporation of these materials broadens the light absorption range, enabling the composite to utilize a larger portion of the solar spectrum, particularly under visible light, which significantly improves its photocatalytic activity. The Z-scheme configuration within this ternary system preserves the high reduction and oxidation potentials of electrons and holes, respectively, enabling the composite to efficiently participate in a wide range of redox reactions, including the degradation of pollutants and water splitting. Moreover, the synergistic effects among Ti_3_C_2_, Bi_2_WO_4_, and LDH not only enhance the composite’s stability and durability under photocatalytic conditions but also ensure sustained activity over extended periods, making the LDH/Ti_3_C_2_/Bi_2_WO_6_ system a highly efficient and versatile option for environmental remediation and energy conversion technologies.

The enhancement of PEC activity in this nanocomposite photoelectrode is the result of multiple synergistic effects, notably the following: (i) The enhanced exposure of {001} facets in Bi_2_WO_6_ nanocomposites within the NiFe-LDH/Ti_3_C_2_/Bi_2_WO_6_ framework significantly contributes to an increased generation of holes. This feature not only facilitates more active participation in the degradation process but also substantially strengthens photocatalytic oxidation reactions. The {001} facets of Bi_2_WO_6_ are known for their high photocatalytic activity, and their prominent exposure within the composite structure maximizes the photocatalytic sites available for reaction; (ii) The strategic integration of NiFe-LDH with Ti_3_C_2_ creates a highly effective conduit for the swift separation and transfer of photogenerated charges. Under illumination, the electrons excited from the conduction band of Bi_2_WO_6_ are quickly transferred to Ti_3_C_2_. This transition is facilitated by the close interfacing and strong electronic interaction between Bi_2_WO_6_ and Ti_3_C_2_, which serves as an effective conduit for the rapid transportation of electrons to the counter electrode via the external circuit. This bridging significantly curtails the recombination of photogenerated electron–hole pairs, thereby enhancing the overall separation efficiency of charge carriers; (iii) The directed migration of photogenerated holes towards the NiFe-LDH surface plays a crucial role in the oxidative degradation of organic pollutants. NiFe-LDH acts as a catalyst that accelerates the oxidation process, converting organic contaminants into carbon dioxide (CO_2_) and water (H_2_O). This process is facilitated by the high affinity of NiFe-LDH for photogenerated holes, which promotes their accumulation on the LDH surface, thereby increasing local hole concentration and enhancing the oxidation reaction rate. As a result, the NiFe-LDH/Ti_3_C_2_/Bi_2_WO_6_ nanocomposite photoelectrode represents a sophisticated integration of materials that leverages the unique properties of each component to maximize PEC degradation efficiency. The optimized exposure of photocatalytically active facets, coupled with the strategic facilitation of charge separation and targeted reaction pathways, underpin the superior performance of this ternary composite in the PEC degradation of organic pollutants. This comprehensive understanding of the degradation mechanism provides valuable insights into designing more efficient PEC systems for environmental remediation.

## 3. Conclusions

In summary, the NiFe-LDH/Ti_3_C_2_/Bi_2_WO_6_ nanocomposite photoelectrode, featuring a significant proportion of {001}-oriented facets, was successfully synthesized through a solvothermal reaction followed by electrochemical deposition. Comparative analysis under electrochemical (EC), photocatalytic (PC), and photoelectrochemical (PEC) conditions revealed that the NiFe-LDH/Ti_3_C_2_/Bi_2_WO_6_ nanocomposite photoelectrode exhibits superior degradation performance relative to the pristine Bi_2_WO_6_ nanocomposite photoelectrode. The activity ranking, in descending order, is as follows: NiFe-LDH/Ti_3_C_2_/Bi_2_WO_6_ NAs > NiFe-LDH/Bi_2_WO_6_ NAs > Ti_3_C_2_/Bi_2_WO_6_ NAs > Bi_2_WO_6_ NAs. The augmented photocatalytic efficacy of the NiFe-LDH/Ti_3_C_2_/Bi_2_WO_6_ nanocomposite photoelectrode can be ascribed to the synergistic interplay between Ti_3_C_2_ and NiFe-LDH, which significantly enhances the charge separation efficiency and reaction kinetics of Bi_2_WO_6_ NAs. This study elucidates a novel approach for enhancing reaction kinetics in the PEC degradation of pollutants.

## 4. Materials and Methods

### 4.1. Preparation of Bi_2_WO_6_ NAs Photoelectrode

Initially, 0.9 g of polyvinylpyrrolidone (PVP) was dissolved in 30 mL of ethylene glycol at a temperature of 95 °C in two separate beakers to achieve a homogeneous, transparent solution. Subsequently, 0.3299 g of sodium tungstate dihydrate (Na_2_WO_4_·2H_2_O) was dissolved in the first beaker (hereafter referred to as solution 1), while 0.9701 g of bismuth nitrate pentahydrate (Bi(NO_3_)_3_·5H_2_O) was dissolved in the second beaker (referred to as solution 2). Solution 1 was then gradually introduced into solution 2 with constant stirring to ensure thorough mixing. The resultant mixture was subjected to a pre-reaction phase at 98 °C for 1.5 h with sustained stirring. Following this, the mixture was transferred into a Teflon-lined autoclave, in which cleaned indium tin oxide (ITO) substrates had been vertically positioned, and subsequently heated at 178 °C for 24 h. Upon cooling to ambient temperature, the ITO substrates were carefully removed, and washed multiple times with ethanol and deionized water to obtain the Bi_2_WO_6_ nanoplate arrays (NAs) photoelectrode.

### 4.2. Preparation of Ti_3_C_2_

A mass of 1.0 g of MAX phase powder (Ti_3_AlC_2_) was dispersed in a Teflon container. To this, 10 mL of 40% HF solution was added per gram of MAX phase, ensuring the mixture was stirred gently to achieve uniform etching. The reaction was allowed to proceed for 24 h at room temperature under constant stirring to facilitate the complete removal of the A layer. Following etching, the mixture was diluted with deionized water and allowed to settle, enabling the separation of undissolved solids. The supernatant, containing excess HF and reaction byproducts, was carefully decanted. This washing process was repeated several times until the pH of the supernatant reached neutrality, indicating the effective removal of residual HF. The resulting MXene was then collected by centrifugation at 3500 rpm for 5 min, washed with deionized water, and spread out for drying at room temperature in a fume hood or under vacuum at 60 °C. The dried MXene was stored in an airtight container under N_2_ atmosphere to prevent oxidation.

### 4.3. Preparation of NiFe-LDH

A volume of 70 mL of deionized water was introduced into an electrolytic cell, followed by the addition of 0.15 mol of nickel nitrate hexahydrate (Ni(NO_3_)_2_·6H_2_O) and 0.15 mol of iron(II) sulfate heptahydrate (FeSO_4_·7H_2_O). An indium tin oxide (ITO) substrate, a platinum wire, and an Ag/AgCl electrode were utilized as the working electrode, counter electrode, and reference electrode, respectively. The electrochemical deposition was conducted at a voltage of −1.0 V for a duration of 300 s. Subsequent to the electrochemical deposition, the ITO substrate was removed and exposed to ambient air to facilitate the natural oxidation of Fe^2+^ ions to Fe^3+^ ions. This process culminated in the formation of the NiFe–layered double hydroxide (LDH) electrode.

### 4.4. Preparation of Ti_3_C_2_/Bi_2_WO_6_ NAs Photoelectrode

Initially, 20 mL of ethylene glycol was introduced into two beakers, and subsequently placed in a 95 °C water bath. Nitrogen gas was infused into each beaker to establish an inert environment. To these, 0.6 g of polyvinylpyrrolidone (PVP) was added as a dispersant. Specifically, beaker 2 received 0.0093 g of Ti_3_C_2_ powder, with nitrogen flow maintained to protect the sensitive material. The mixture was sealed and magnetically stirred for 20 min, ensuring uniform dispersion of Ti_3_C_2_.

Simultaneously, 0.3299 g of Na_2_WO_4_·2H_2_O and 0.9701 g of Bi(NO_3_)3·5H_2_O were added to beakers 1 and 2, respectively, under nitrogen to prevent any oxidative reactions. The contents of beaker 1 were slowly added to beaker 2, and the combined solution was pre-reacted for one hour at 95 °C, still under nitrogen. This mixture was then transferred to a 50 mL autoclave containing a pre-treated indium tin oxide (ITO) substrate, with the entire assembly purged with nitrogen. The autoclave underwent hydrothermal treatment at 180 °C for 24 h, facilitating formation of the composite on the ITO, all while under nitrogen to minimize degradation. Upon cooling to room temperature under nitrogen, the ITO substrate, coated with the Ti_3_C_2_/Bi_2_WO_6_ composite, was meticulously rinsed with deionized water and anhydrous ethanol, and dried in a vacuum oven at 60 °C.

### 4.5. Preparation of NiFe-LDH/Bi_2_WO_6_ NAs Photoelectrode

A volume of 70 mL of deionized water was added to an electrolytic cell. Then, under a nitrogen atmosphere and with continuous stirring, 0.15 M of Ni(NO_3_)2·6H_2_O and 0.15 M of FeSO_4_·7H_2_O were added, respectively. The flow of N_2_ gas was to prevent the oxidation of Fe^2+^. The electrochemical deposition of NiFe-LDH was carried out using a three-electrode system. A Bi_2_WO_6_ photoelectrode, a platinum wire, and an Ag/AgCl electrode were used as the working electrode, counter electrode, and reference electrode, respectively. The deposition voltage was set to −1.0 V, and the deposition time was 30 *s*. After the electrochemical deposition finished, the ITO was removed, rinsed with deionized water, and then placed in air to allow Fe^2+^ to naturally oxidize to Fe^3+^. Finally, a NiFe-LDH/Bi_2_WO_6_ or NiFe-LDH/Ti_3_C_2_/Bi_2_WO_6_ composite photoelectrode was obtained.

### 4.6. Preparation of NiFe-LDH/Ti_3_C_2_/Bi_2_WO_6_ NAs Photoelectrode

A Ti_3_C_2_/Bi_2_WO_6_ NAs photoelectrode was used as the working electrode, and the experimental steps were repeated as in Section 4.5.

### 4.7. Characterization Techniques

X-ray diffraction (XRD) analyses were conducted using a Bruker D8-Focus diffractometer equipped with Cu Kα radiation. The morphology of the synthesized sample was examined via a scanning electron microscope (SEM, SU8010, Hitachi Ltd., Chiyoda, Japan) across an acceleration voltage range of 200 V to 30 kV, supplemented by energy dispersive X-ray (EDX) spectroscopy for elemental analysis. The interplanar spacing of the lattice fringes was determined using a high-resolution transmission electron microscope (HRTEM, Tecnai F20, FEI Company, Hillsboro, OR, USA). Ultraviolet-visible diffuse reflectance spectra (DRS) were acquired on a Hitachi 4100 spectrophotometer, employing BaSO_4_ as the reference standard. X-ray photoelectron spectroscopy (XPS) measurements were performed on a PHI Quantera ULVAC XPS system (ULVAC, Inc., Chigasaki, Japan). Photoelectrocatalytic (PEC) characterizations were conducted in a quartz electrochemical cell, incorporating a three-electrode setup, with an electrochemical workstation (CHI 660D, CH Instruments, Inc., Shanghai, China). A 0.1 M solution of Na_2_SO_4_ served as the electrolyte. Electrochemical impedance spectroscopy (EIS) was executed over a frequency range from 0.005 Hz to 105 Hz, applying a sinusoidal AC disturbance signal of 5 mV to probe the electrochemical properties of the materials.

### 4.8. Degradation Activity Test

The PC and PEC activities of the synthesized samples were assessed through the degradation of bisphenol A (BPA) at a concentration of 10 mol·L^−1^ in a 50 mL solution of Na_2_SO_4_ (0.1 mol·L^−1^). Prior to initiating the PC and PEC reactions, the solution was stirred in darkness for 30 min to establish adsorption–desorption equilibrium. For the PEC activity evaluation, a photoelectrode, a Pt wire, and a saturated calomel electrode (SCE) were employed as the working electrode, counter electrode, and reference electrode, respectively. The photoelectrode was exposed to either simulated sunlight or visible light (λ > 420 nm), generated by a 300 W Xe lamp (PLS-SXE300C/300CUV, Perfect Light Ltd, Beijing, China), with an average intensity of 100 mW cm^−2^. For comparative analysis, the samples were also subjected to electrochemical (EC) degradation of BPA under identical conditions but without light irradiation. The degradation process was conducted over a duration of 4 h, with aliquots of 2.5 mL sampled every 30 min. The concentration of BPA was determined using a high-performance liquid chromatography (HPLC) system (Shimadzu LC-20AT, Shimadzu Corporation, Kyoto, Japan).

## Data Availability

Data supporting the findings of this study are available from the corresponding author upon reasonable request.

## Supplementary Materials

The following supporting information can be downloaded at https://www.mdpi.com/article/10.3390/nano14050477/s1: Figure S1. SEM image of Bi_2_WO_6_ NAs; Figure S2. EDX mapping of NiFe-LDH/Ti_3_C_2_/Bi_2_WO_6_ NAs; Figure S3. Comparison of EC, PC, and PEC degradation rates over Bi_2_WO_6_ NAs, NiFe-LDH/Bi_2_WO_6_ NAs, Ti_3_C_2_/Bi_2_WO_6_ NAs, and NiFe-LDH/Ti_3_C_2_/Bi_2_WO_6_ NAs (λ ≥ 420 nm, external potential = 1.0 V); Figure S4. Comparison of the PEC degradation rate of BPA over NiFe-LDH/Ti_3_C_2_/Bi_2_WO_6_ NAs at different bias voltages (λ ≥ 420 nm); Figure S5. Photoelectric conversion efficiency of Bi_2_WO_6_ NAs, NiFe-LDH/Bi_2_WO_6_ NAs, Ti_3_C_2_/Bi_2_WO_6_ NAs, and NiFe-LDH/Ti_3_C_2_/Bi_2_WO_6_ NAs.
