# Supplementary material for: LDH/MXene Synergistic Carrier Separation Effects to Improve the Photoelectric Catalytic Activities of Bi2WO6 Nanosheet Arrays
Source: Nanomaterials (Basel). 2024 Mar 6;14(5):477. doi: 10.3390/nano14050477 (PMC10935222; doi:10.3390/nano14050477)
Supplement: Supplementary file 1 [file nanomaterials-14-00477-s001.zip › nanomaterials-2865639-supplementary.pdf]

nanomaterials

MDPI

Article

LDH/MXene Synergistic Carrier Separation Effects to Improve the Photoelectric Catalytic Activities of Bi_2WO_6 Nanosheet Arrays

Yuting Wang †, Runhua Li †, Jiaying Zhang, Liming Liu, Weiwei Huang and Yajun Wang *

State Key Laboratory of Heavy Oil Processing, China University of Petroleum, Beijing 102249, China; wangyt@cup.edu.cn (Y.W.); 2021215954@student.cup.edu.cn (R.L.); 2020211160@student.cup.edu.cn (J.Z.); 2021211158@student.cup.edu.cn (L.L.); 2022211152@student.cup.edu.cn (W.H.)

* Correspondence: wangyajun@cup.edu.cn

† These authors contributed equally to this work.

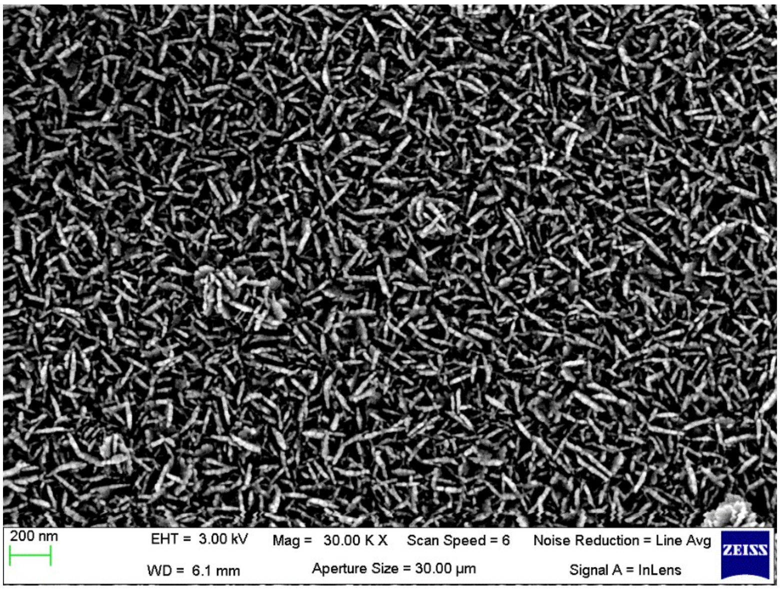

Figure S1. SEM image of Bi_2WO_6 NAs.

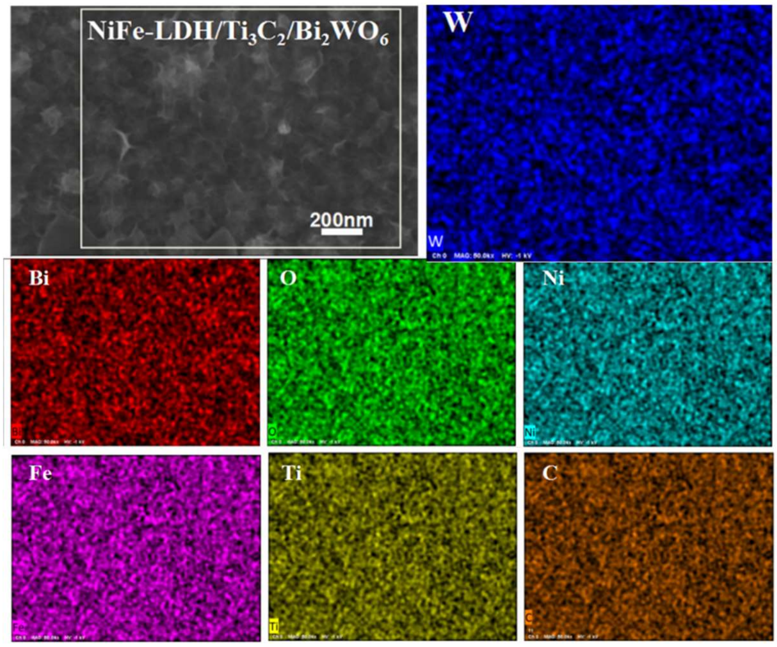

Figure S2. EDX mapping of NiFe-LDH/Ti_3C_2/Bi_2WO_6 NAs.

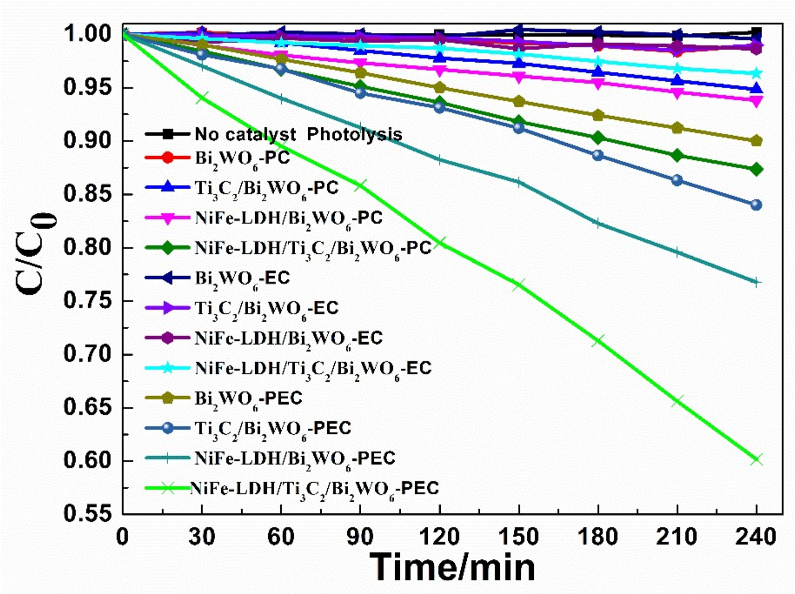

Figure S3. Comparison of EC, PC, and PEC degradation rates over Bi_2WO_6 NAs, NiFe-LDH/Bi_2WO_6 NAs, Ti_3C_2/Bi_2WO_6 NAs and NiFe-LDH/Ti_3C_2/Bi_2WO_6 NAs ($\lambda \geq$ 420nm, external potential= 1.0 V).

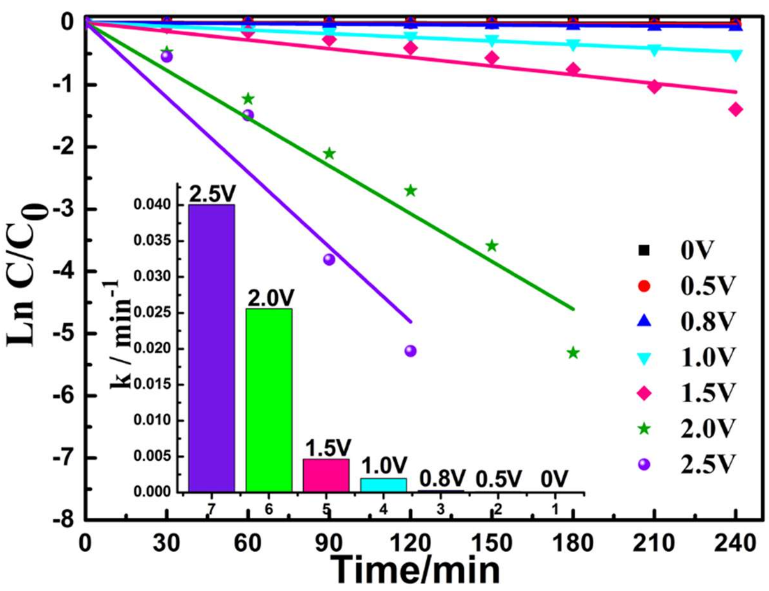

Figure S4. Comparison of the PEC degradation rate of BPA over NiFe-LDH/Ti_3C_2/Bi_2WO_6 NAs at different bias voltage (λ≥420 nm).

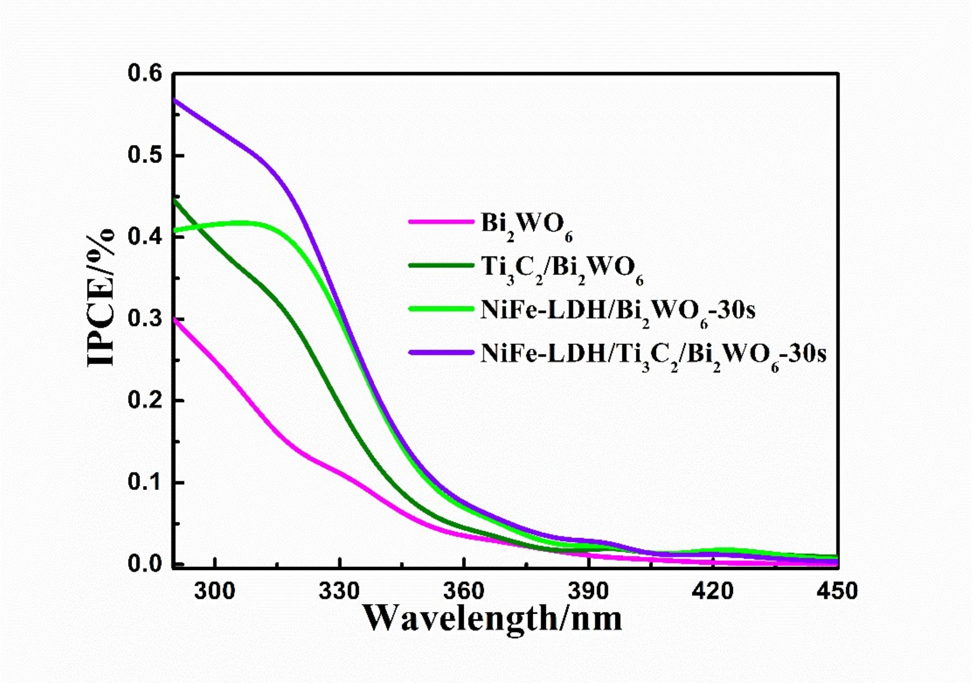

Figure S5. Photoelectric conversion efficiency of Bi_2WO_6 NAs, NiFe-LDH/Bi_2WO_6 NAs, Ti_3C_2/Bi_2WO_6 Nas, and NiFe-LDH/Ti_3C_2/Bi_2WO_6 NAs.
